# ClassyFire: automated chemical classification with a comprehensive, computable taxonomy

**DOI:** 10.1186/s13321-016-0174-y

**Published:** 2016-11-04

**Authors:** Yannick Djoumbou Feunang, Roman Eisner, Craig Knox, Leonid Chepelev, Janna Hastings, Gareth Owen, Eoin Fahy, Christoph Steinbeck, Shankar Subramanian, Evan Bolton, Russell Greiner, David S. Wishart

**Affiliations:** 1 Department of Biological Sciences, University of Alberta, Edmonton, AB T6G 2E8 Canada; 2 Jobber – Field Service Software, 10520 Jasper Ave, Edmonton, AB T5J 1Z7 Canada; 3 Department of Computing Science, University of Alberta, Edmonton, AB T6G 2E8 Canada; 4 National Research Council, National Institute for Nanotechnology (NINT), Edmonton, AB T6G 2M9 Canada; 5 Department of Medical Imaging, The Ottawa Hospital, University of Ottawa, Civic Campus, 1053 Carling Ave, Ottawa, ON K1Y 4E9 Canada; 6 European Molecular Biology Laboratory - European Bioinformatics Institute (EMBL-EBI), Wellcome Trust Genome Campus, Hinxton, Cambridge UK; 7 Department of Bioengineering, University of California, La Jolla, San Diego, CA 92093 USA; 8 Department of Health and Human Services, National Center for Biotechnology Information, National Library of Medicine, National Institutes of Health, 8600 Rockville Pike, Bethesda, MD 20894 USA; 9 Department of Computing Science 2-21 Athabasca Hall, Alberta Innovates Centre for Machine Learning (AICML), University of Alberta, Edmonton, AB T6G 2E8 Canada; 10 The Metabolomics Innovation Center, University of Alberta, Edmonton, AB T6G 2E9 Canada

**Keywords:** Structure-based classification, Ontology, Taxonomy, Text-based search, Inference, Annotation, Database, Data integration

## Abstract

**Background:**

Scientists have long been driven by the desire to describe, organize, classify, and compare objects using taxonomies and/or ontologies. In contrast to biology, geology, and many other scientific disciplines, the world of chemistry still lacks a standardized chemical ontology or taxonomy. Several attempts at chemical classification have been made; but they have mostly been limited to either manual, or semi-automated proof-of-principle applications. This is regrettable as comprehensive chemical classification and description tools could not only improve our understanding of chemistry but also improve the linkage between chemistry and many other fields. For instance, the chemical classification of a compound could help predict its metabolic fate in humans, its druggability or potential hazards associated with it, among others. However, the sheer number (tens of millions of compounds) and complexity of chemical structures is such that any manual classification effort would prove to be near impossible.

**Results:**

We have developed a comprehensive, flexible, and computable, purely structure-based chemical taxonomy (ChemOnt), along with a computer program (ClassyFire) that uses only chemical structures and structural features to automatically assign all known chemical compounds to a taxonomy consisting of >4800 different categories. This new chemical taxonomy consists of up to 11 different levels (Kingdom, SuperClass, Class, SubClass, etc.) with each of the categories defined by unambiguous, computable structural rules. Furthermore each category is named using a consensus-based nomenclature and described (in English) based on the characteristic common structural properties of the compounds it contains. The ClassyFire webserver is freely accessible at http://classyfire.wishartlab.com/. Moreover, a Ruby API version is available at https://bitbucket.org/wishartlab/classyfire_api, which provides programmatic access to the ClassyFire server and database. ClassyFire has been used to annotate over 77 million compounds and has already been integrated into other software packages to automatically generate textual descriptions for, and/or infer biological properties of over 100,000 compounds. Additional examples and applications are provided in this paper.

**Conclusion:**

ClassyFire, in combination with ChemOnt (ClassyFire’s comprehensive chemical taxonomy), now allows chemists and cheminformaticians to perform large-scale, rapid and automated chemical classification. Moreover, a freely accessible API allows easy access to more than 77 million “ClassyFire” classified compounds. The results can be used to help annotate well studied, as well as lesser-known compounds. In addition, these chemical classifications can be used as input for data integration, and many other cheminformatics-related tasks.

**Electronic supplementary material:**

The online version of this article (doi:10.1186/s13321-016-0174-y) contains supplementary material, which is available to authorized users.

## Background

Taxonomies and ontologies organize complex knowledge about concepts and their relationships. Biology was one of the first fields to use these concepts. Taxonomies are simplistic schemes that help in the hierarchical classification of concepts or objects [1]. They are usually limited to a specific domain and to a single relationship type connecting one node to another. Ontologies share the hierarchical structure of taxonomies. In contrast to taxonomies, however, they often have multiple relationship types and are really designed to provide a formal naming of the types, properties and interrelationships of entities or concepts in a specific discipline, domain or field of study [2, 3]. Moreover, ontologies provide a system to create relationships between concepts across different domains. Both taxonomies and ontologies can be used to help scientists explain, organize or improve their understanding of the natural world. Furthermore, taxonomies and ontologies can serve as standardized vocabularies to help provide inference/reasoning capabilities. In fact, taxonomies and ontologies are widely used in many scientific fields, including biology (the *Linnean* taxonomy) [4], geology (the BGS Rock classification scheme) [5], subatomic physics (the Eightfold way) [6], astronomy (the stellar classification system) [7, 8] and pharmacology (the ATC drug classification system) [9]. One of the most widely used ontologies is the Gene Ontology (GO) [10], which serves to annotate genes and their products in terms of their molecular functions, cellular locations, and biological processes. Given a specific enzyme, such as the human cytosolic phospholipase (PLA2G4A), and its GO annotation, one could infer the cellular location of its substrate PC[14:0/22:1(13Z)] (HMDB07887). Additionally, because PLA2G4A is annotated with the GO term “phospholipid catabolic process”, it could be inferred that PC[14:0/22:1(13Z)] is a product of this biological process.

While chemists have been very successful in developing a standardized nomenclature (IUPAC) and standardized methods for drawing or exchanging chemical structures [11, 12], the field of chemistry still lacks a standardized, comprehensive, and clearly defined chemical taxonomy or chemical ontology to robustly characterize, classify and annotate chemical structures. Consequently, chemists from various chemistry specializations have often attempted to create domain-specific ontologies. For instance, medicinal chemists tend to classify chemicals according to their pharmaceutical activities (antihypertensive, antibacterials) [9], whereas biochemists tend to classify chemicals according to their biosynthetic origin (leukotrienes, nucleic acids, terpenoids) [13]. Unfortunately, there is no simple one-to-one mapping for these different classification schemes, most of which are limited to very small numbers of domain-specific molecules. Thus, the last decade has seen a growing interest in developing a more universal chemical taxonomy and chemical ontology.

**Fig. 1:**
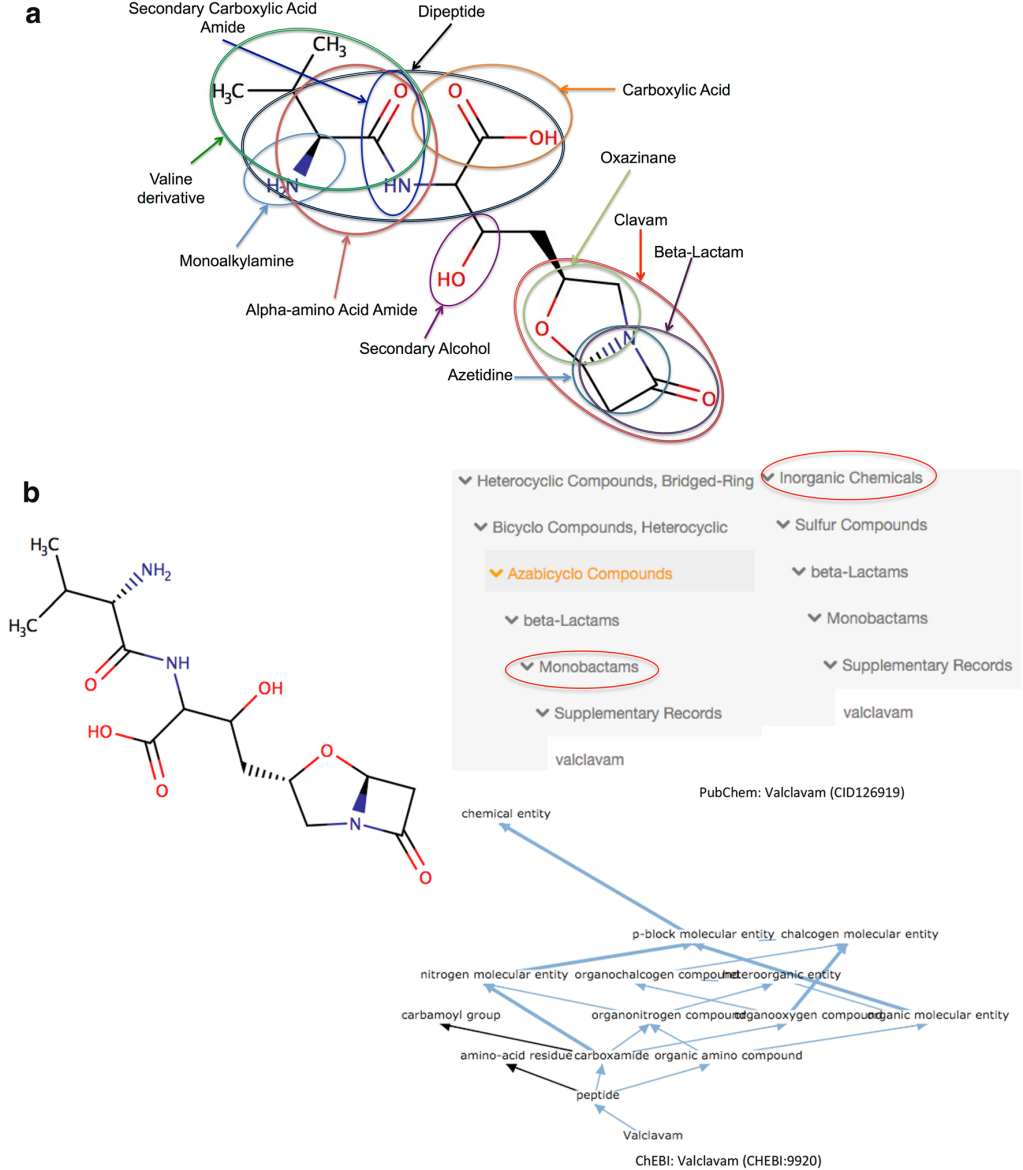
**a** Valclavam is annotated in the PubChem (CID 126919) and ChEBI (CHEBI:9920) databases. **b** In PubChem, it is incorrectly assigned the class of beta-lactams, which are sulfur compounds. Moreover, although the latter can be either inorganic or organic, it is wrong to describe a single compound both as organic and inorganic. The transitivity of the *is_a* relationship is not fulfilled, which makes the class inference difficult. In ChEBI, the same compound is correctly classified as a peptide. However, as in PubChem, the annotation is incomplete. Class assignments to “clavams” and “azetidines”, among others, are missing

To date, most attempts aimed at classifying and describing chemical compounds have been structure-based. This is largely because the bioactivity of a compound is influenced by its structure [14]. Moreover, the structure of a compound can be easily represented in various formats. Some examples of structure-based chemical classification or ontological schemes include the ChEBI ontology [15], the Medical Subject Heading (MeSH) thesaurus [16], and the LIPID MAPS classification scheme [13]. These databases and ontologies/thesauri are excellent and have been used in various studies including chemical enrichment analysis [17], and knowledge-based metabolic model reconstruction [18], among others. However, they are all produced manually, thus making the classification/annotation process somewhat tedious, error-prone and inconsistent (Fig. 1). In addition, they require substantial human expert time, which means these classification systems only cover a tiny fraction of known chemical space. For instance, in the PubChem database [19], only 0.12% of the >91,000,000 compounds (as of June 2016) are actually classified via the MeSH thesaurus.

There are several other, older or lesser-known chemical classification schemes, ontologies or taxonomies that are worth mentioning. The Chemical Fragmentation Coding system [20] is perhaps the oldest taxonomy or chemical classification scheme. It was developed in 1963 by the Derwent World Patent Index (DWPI) to facilitate the manual classification of chemical compounds reported in patents. The system consists of 2200 numerical codes corresponding to a set of pre-defined, chemically significant structure fragments. The system is still used by Derwent indexers who manually assign patented chemicals to these codes. However, the system is considered outdated and complex. Likewise, using the chemical fragmentation codes requires practice and extensive guidance of an expert. A more automated alternate to the Derwent index was developed in the 1970s, called the HOSE (Hierarchical Organisation of Spherical Environments) code [21]. This hierarchical substructure system, allows one to automatically characterize atoms and complete rings in terms of their spherical environment. It employs an easily implemented algorithm that has been widely used in NMR chemical shift prediction. However, the HOSE system does not provide a named chemical category assignment nor does it provide an ontology or a defined chemical taxonomy. More recently, the Chemical Ontology (CO) system [22] has been described. Designed to be analogous to the Gene Ontology (GO) system, CO was one of the first open-source, automated functional group ontologies to be formalized. CO functional groups can be automatically assigned to a given structure by Checkmol [23], a freely available program. CO’s assignment of functional groups is accurate and consistent, and it has been applied to several small datasets. However, the CO system is limited to just ~200 chemical groups, and so it only covers a very limited portion of chemical space. Moreover, Checkmol is very slow and is impractical to use on very large data sets. SODIAC [24] is another promising tool for automatic compound classification. It uses a comprehensive chemical ontology and an elegant structure-based reasoning logic. SODIAC is a well-designed commercial software package that permits very rapid and consistent classification of compounds. The underlying chemical ontology can be freely downloaded and the SODIAC software, which is closed-source, is free for academics. The fact that it is closed-source obviously limits the possibilities for community feedback or development. Moreover, the SODIAC ontology does not provide textual definitions for most of its terms and is limited in its coverage of inorganic and organo-metallic compounds. Other notable efforts directed towards chemical classification or clustering include Maximum Common Substructure (MCS) based methods [25, 26], an iterative scaffold decomposition method introduced by Shuffenhauer et al. [27], and a semantic-based method described by Chepelev et al. [28]. However, most of these are proof-of-principle methods and have only been validated on a small number of compound classes, which cover only a tiny portion of rich chemical space. Moreover, they are very data-set dependent. As a result, the classifications do not match the nomenclature expectations of the chemical community, especially for complex compound classes.

Overall, it should be clear that while many attempts have been made to create chemical taxonomies or ontologies, many are proprietary or “closed source”, most require manual analysis or annotation, most are limited in scope and many do not provide meaningful names, definitions or descriptors. These shortcomings highlight the need to develop open access, open-source, fast, fully automated, comprehensive chemical classification tools with robust ontologies that generate results that match chemists’ (i.e. domain experts’) and community expectations. Furthermore, such tools must rapidly classify chemical entities in a consistent manner that is independent of the type of chemical entity being analyzed.

The development of a fully automated, comprehensive chemical classification tool also requires the use of a well-defined chemical hierarchy, whether it is a taxonomy or an ontology. This means that the criteria for hierarchy construction, the relationship types, and the scope of the hierarchy must be clearly defined. Additionally, a clear set of classification rules and a comprehensive data dictionary (or ontology) are necessary. Furthermore, comprehensive chemical classification requires that the chemical categories present in the taxonomy/ontology must be accurately described in a computer-interpretable format. Because new chemical compounds and new “chemistries” are being developed or discovered all the time, the taxonomy/ontology must be flexible and any extension should not force a fundamental modification of the classification procedure. In this regard, Hasting et al. [29] suggested a list of principles that would facilitate the development of an intelligent chemical structure-based classification system. One of the main criteria in this schema is the possibility to combine different elementary features into complex category definitions using compositionality. This is very important, since chemical classes are structurally diverse. Additionally, an accurate description of their core structures sometimes requires the ability to express constraints such as substitution patterns. Today, this can be achieved to a certain extent by the use of logical connectives and structure-handling technologies such as the SMiles ARbitrary Target Specification (SMARTS) format.

In this paper, we describe a comprehensive, flexible, computable, chemical taxonomy along with a fully annotated chemical ontology (ChemOnt) and a Chemical Classification Dictionary. These components underlie a web-accessible computer program called ClassyFire, which permits automated rule-based structural classification of essentially all known chemical entities. ClassyFire makes use of a number of modern computational techniques and circumvents most of the limitations of the previously mentioned systems and software tools. This paper also describes the rationale behind ClassyFire, its classification rules, the design of its taxonomy, its performance under testing conditions and its potential applications. ClassyFire has been successfully used to classify and annotate >6000 molecules in DrugBank [30], >25,000 molecules in the LIPID MAPS Lipidomics Gateway [31], >42,000 molecules in HMDB [32], >43,000 compounds in ChEBI [15] and >60,000,000 molecules in PubChem [19], among others. These compounds cover a wide range of chemical types such as drugs, lipids, food compounds, toxins, phytochemicals and many other natural as well as synthetic molecules. ClassyFire is freely available at http://classyfire.wishartlab.com. Moreover, the ClassyFire API, which is written in Ruby, provides programmatic access to the ClassyFire server and database. It is available at https://bitbucket.org/wishartlab/classyfire_api.

## Methods

Creating a computable chemical taxonomy requires three key components: (1) a well-defined hierarchical taxonomic structure; (2) a dictionary of chemical classes (with full definitions and category mappings); and (3) computable rules or algorithms for assigning chemicals to taxonomic categories. Each of these components is described in more detail below.

### Component 1—Hierarchical taxonomic structure

A taxonomy requires a well-defined, structured hierarchy. Following standard notation, we use the term “category” to refer to any chemical class (at any level), each of which corresponds to a set of chemicals. These categories are arranged in a tree structure (Additional file 1). The main relationship type connecting these different categories is the “*is_a*” relationship. The rationale behind the choice of a tree structure was to provide a detailed annotation represented via a simple data structure, which could be easily understandable by humans. Moreover, as described in the results section, ClassyFire provides a list of all parents of a compound, which makes it easy to infer all of its ancestors. Inspired by the original Linnaean biological taxonomy [4], we assigned the terms Kingdom, SuperClass, Class, and SubClass to denote the first, second, third and fourth levels of the chemical taxonomy, respectively. The top level (Kingdom) partitions chemicals into two disjoint categories: organic compounds versus inorganic compounds. Organic compounds are defined as chemical compounds whose structure contains one or more carbon atoms. Inorganic compounds are defined as compounds that are not organic, with the exception of a small number of “special” compounds, including, cyanide/isocyanide and their respective non-hydrocarbyl derivatives, carbon monoxide, carbon dioxide, carbon sulfide, and carbon disulfide. For the complete current list of exceptions, please see Additional file 1. The classification of compounds into these two kingdoms aligns with most modern views of chemistry and is easily performed on the basis of a compound’s molecular formula. The other levels in our classification schema depend on much more detailed definitions and rules that are described below. SuperClasses (which includes 26 organic and 5 inorganic categories) consist of generic categories of compounds with general structural identifiers (e.g. organic acids and derivatives, phenylpropanoids and polyketides, organometallic compounds, homogeneous metal compounds), each of which covers millions of known compounds. The next level below the SuperClass level is the Class level, which now includes 764 nodes. Classes typically consist of more specific chemical categories with more specific and recognizable structural features (pyrimidine nucleosides, flavanols, benzazepines, actinide salts). Chemical Classes usually contain >100,000 known compounds. The level below Classes represents SubClasses, which typically consist of >10,000 known compounds. There are 1729 SubClasses in the current taxonomy. Additionally, there are 2296 additional categories below the SubClass level covering taxonomic levels 5–11.

**Fig. 2:**
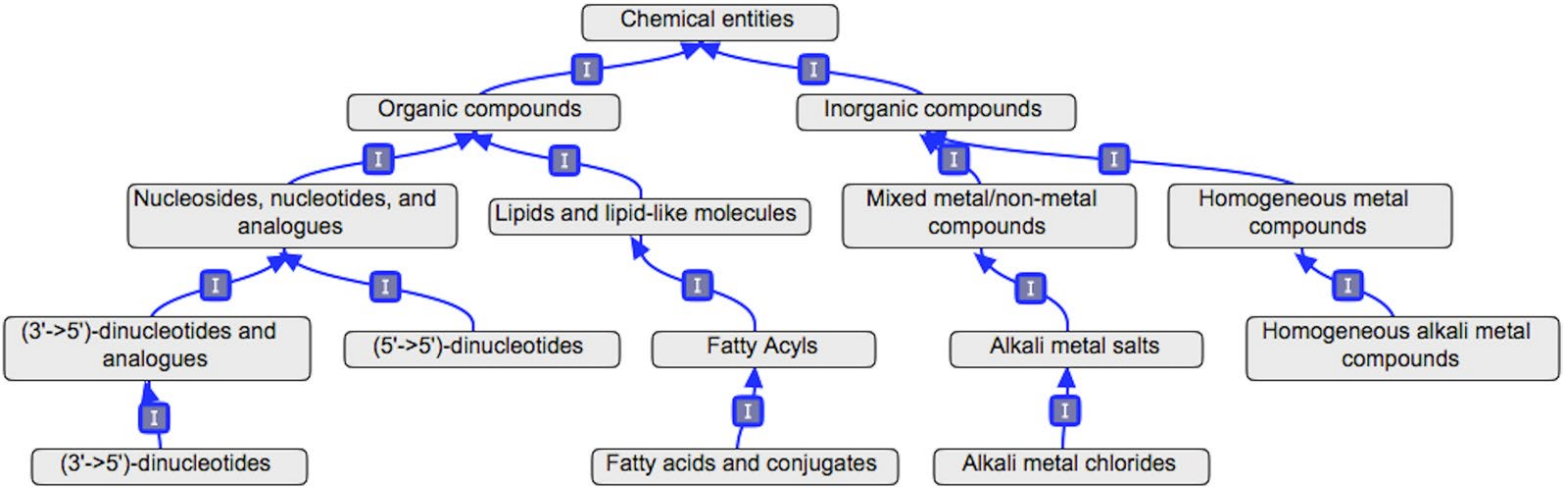
Illustration of the taxonomy as a tree

Altogether this extensive chemical taxonomy contains a total of 4825 chemical categories of organic (4146) and inorganic (678) compounds, in addition to the root category (Chemical entities). As a whole, this chemical taxonomy can be represented as a tree with a maximum depth of 11 levels, and an average depth of five levels per node (Fig. 2). As with any structured taxonomy, the creation of a well-defined hierarchical structure offers the possibility to focus on a sub-domain of the chemical space, or a specific level of classification. A more complete description of this taxonomic hierarchy can be found in the Additional file 1: Table S1. The chemical taxonomy and its hierarchical structure provided using the Open Biological and Biomedical Ontologies (OBO) format [33], which may help with its integration with respect to semantic technology approaches. The resulting OBO file was generated with OBO-Edit [34], and can be downloaded from the ClassyFire website.

### Component 2—Chemical class dictionary

Each node or category name in ClassyFire’s chemical ontology or ChemOnt, was created by extracting common or existing chemical classification category terms from the scientific literature and available chemical databases. We used existing terms to avoid “reinventing the wheel”. By making use of commonly recognized or widely used terms that already exist in the chemical literature, we believed that the taxonomy (and the corresponding ontology) should be more readily adopted and understood. This dictionary creation process was iterative and required the manual review of a large number of specialized chemical databases, textbooks and chemical repositories. Because the same compounds can often be classified into multiple categories, an analysis of the specificity of each categorical term was performed. Those terms that were determined to be clearly generic (e.g. organic acid, organoheterocyclic compound) or described large numbers of known compounds were assigned to SuperClasses. Terms that were highly specific (e.g. alpha-imino acid or derivatives, yohimbine alkaloids) or which described smaller numbers of compounds that clearly fell within a larger SuperClass were assigned to Classes or SubClasses. This assignment also depended on their relationship to higher-level categories. In some cases multiple, equivalent terms were used to describe the same compounds or categories (imidazolines vs. dihydroimidazoles). To resolve these disputes, the frequency with which the competing terms were used was objectively measured (using Google page statistics or literature count statistics). Those having the highest frequency would generally take precedence. However, attention was also paid to the scientific community and expert panels. When available, the IUPAC term was used to name a specific category. Otherwise, if the experts clearly recommended a set of (less frequently used) terms, these would take precedence over terms initially chosen by our initial “popularity” selection criteria. Examples include the terms “Imidazolines” (229,000 Google hits) and “Dihydroimidazoles” (4590 Google hits). The other popular terms were then added as synonyms. A total of 9012 English synonyms were added to the ChemOnt terminology data set.

In a number of cases, new SuperClass and Class terms were created for chemical categories not explicitly defined in the literature. Of these, the resulting “novel” categories were typically constructed from the IUPAC nomenclature for organic and inorganic compounds. Because our chemical dictionary was built from extant or common terms, it contains many community-specific categories commonly used in the (bio-)chemical nomenclature (e.g. primary amines, steroids, nucleosides). Moreover, due to the diverse nature of active and biologically interesting compounds, many chemical categories linked to specific chemical activities or based on biomimetic skeletons (e.g. alpha-sulfonopeptides, piperidinylpiperidines) were added. For instance, several compounds from the category of imidazo[1,2-a]pyrimidines (CHEMONTID:0004377) have been shown to display GABA(A) antagonist activity, and a potential to treat anxiety disorders [35].

**Fig. 3:**
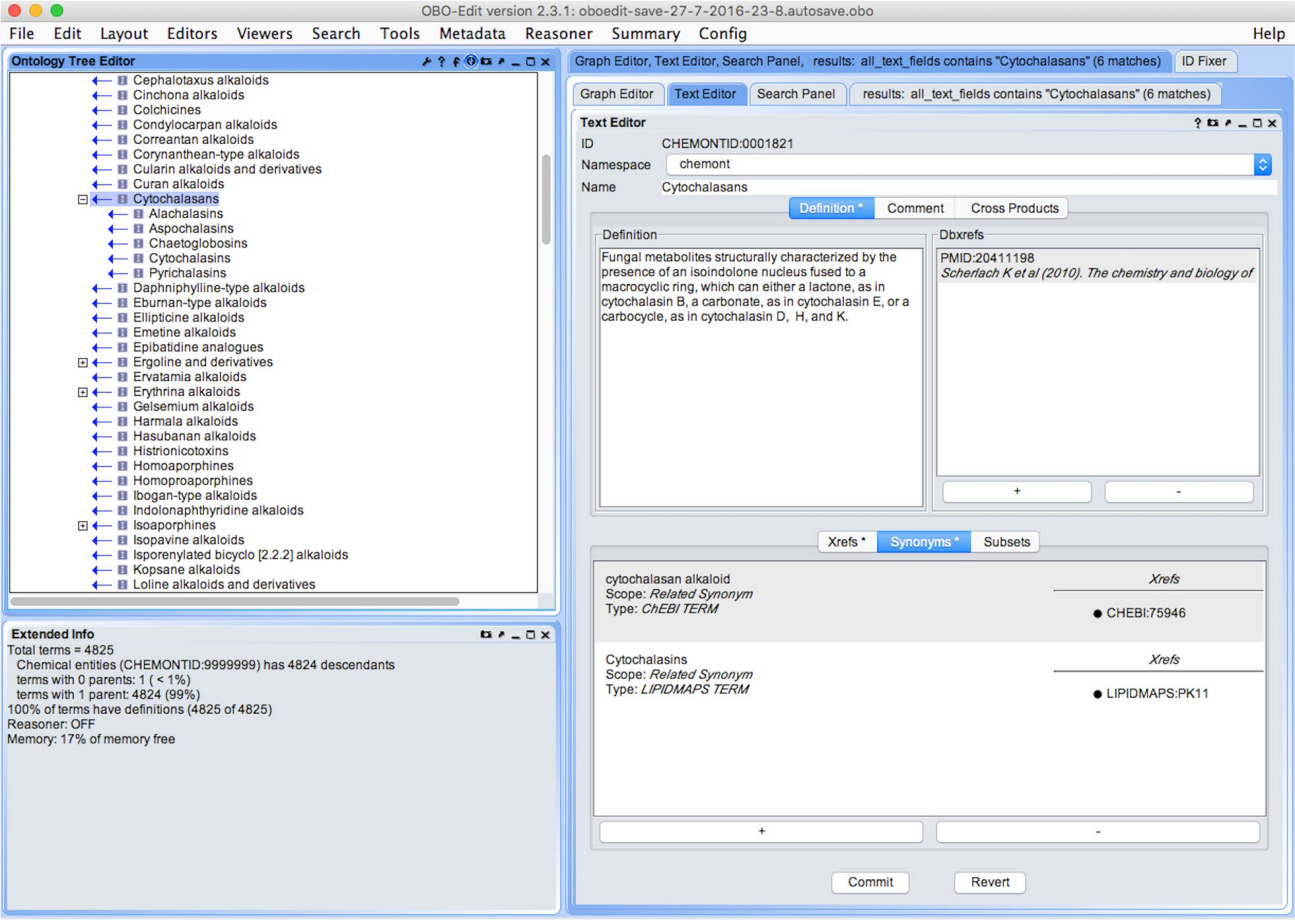
The chemical taxonomy. The taxonomy is illustrated with the OBO-Edit software, showing definitions synonyms, references, and extended information

After all the dictionary terms were identified and compiled (4825 terms to date), each term was formally defined using a precise, yet easily understood text description that included the structural features corresponding to that chemical category (Fig. 3). These formal definitions and the corresponding category mappings formed the basis of the structural classification algorithm and the classification rules described below. Once defined, the terms in this Chemical Classification Dictionary were progressively added to the taxonomic structure to form the structure-based hierarchy underlying ClassyFire’s chemical classification scheme. With the combination of the taxonomic structure and the Chemical Classification Dictionary, ChemOnt can be formally viewed as an ontology (albeit purely a structural ontology).

### Component 3—The classification algorithm

The essence of our classification algorithm is to use the structural definitions and terms contained in the Chemical Classification Dictionary to classify compounds. This required converting the English text definitions into a computable set of rules with each definition consisting of one or more chemical structures, and/or a set of characteristic features that can be otherwise expressed in a computable form. The main format used for chemical structure representation in our classification algorithm is the SMARTS format [28]. SMARTS is a molecular pattern matching language, related to the popular SMILES molecular language, that can be used to specify sub-structural patterns in molecules. For instance, thiazoles are heterocyclic compounds containing a five-member aromatic ring made up of one sulfur atom, one nitrogen, and three carbon atoms. This category of compounds can be described with the following SMARTS expression:

$$[\$ ([\# 16] - 1 - [\# 6] = [\# 6] - [\# 6] = [\# 7] - 1),\$ ([\# 16] - 1 - [\# 6] = [\# 6] - [\# 7] = [\# 6] - 1)]$$

Converting the 4825 definitions in our Chemical Classification Dictionary led to the creation of >9000 SMARTS strings. The validity of each SMARTS string was first tested by performing a superstructure search on small sets of positive or negative example compounds. In most cases, manually generated SMARTS strings, or combinations thereof, were sufficient to represent the vast majority of chemical categories (Additional file 2: Figure S1). However, in some cases, SMARTS strings could not express specific constraints that a given compound must fulfill in order to be assigned a given category. For instance, SMARTS strings cannot describe structures with variable numbers of a specific bond or a specific atom. One way around this would be to enumerate the different patterns, which could easily lead to a combinatorial explosion. For these exceptions we used the Markush format [36], which is available through ChemAxon’s Marvin tool. With the Markush format, it is possible to represent substituent’s variations, position’s variations, as well as the frequency variation of structural groups within a chemical structure. The Markush patterns used by ClassyFire constitute only about 4% of the set of patterns in the ClassyFire database. In addition, some chemical categories were more appropriately defined by a combination of logical expressions based on features such as structural patterns, physico-chemical properties or chemical formulae (Additional file 2: Figure S2). For example, an alkane, which is an acyclic branched or unbranched hydrocarbon having the general formula C_n_H_2n+2_, can be formally represented as the following combination of rules:

$$RingCount\left( A \right) = 0 \wedge AtomCount\left( {C,A} \right) > 0 \wedge \left( {AtomCount\left( {C,A} \right) + AtomCount\left( {H,A} \right) = TotalAtomCount\left( A \right)} \right) \wedge \left( {AtomCount\left( {H,A} \right) = 2 \times AtomCount\left( {C,A} \right) + 2} \right),$$

where *AtomCount*(*X*,*A*) is the number of atoms of type X in the molecule A, *RingCount*(*A*) is the total number of rings in the compound A, and *TotalAtomCount*(*A*) is the total number of atoms in the compound A. In rare cases, some categories of compounds could not be accurately described in an explicit and formal way using any SMARTS string, Markush representation, structural pattern, physico-chemical property or chemical formula. These included certain categories of lipids and lipid-like molecules, phenylpropanoids, polyketides, peptidomimetics and alkaloids, among others. In these cases, the categories were defined as a union of their subcategories that were formally expressed.

It is also important to remember that chemicals can exist as structural chimeras or combinations of different, covalently linked chemical structures, building blocks or domains. Consequently some chemicals (Fig. 1) could potentially belong to more than one chemical class or category. To simplify the chemical classification process, we chose to prioritize the category corresponding to the largest or most dominant structural feature of the chemical compounds (see below). This decision was based on the observed and historical tendencies of chemists to manually classify compounds based on the size (i.e. the number of atoms) of the most dominant structural feature. Furthermore, identifying the largest feature is a technique that is easily measurable and completely objective. If two or more dominant structural features are equal in size, methods described later are used to select one of the features. In ClassyFire’s algorithm, if a structural feature is a represented by structure, its feature weight is equivalent to the number of non-hydrogen atoms in that substructure. If a structural feature is represented by a combination of logical terms, its weight is the total number of non-hydrogen atoms of the smallest compound that fulfills the defined constraints.

It is important that any automated classification tool provide a result that is identical or near-identical to the outcome of manual assignments by experts. As a result, a small number of post hoc adjustments were made for certain well-known chemical categories that are commonly identified by their biochemical context. For instance, we created a category called “Phenylpropanoids and polyketides”. Phenylpropanoids and polyketides can be described as small organic compounds that are synthesized either from the amino acid phenylalanine (phenylpropanoids) or the decarboxylative condensation of malonyl-CoA (polyketides). These classes are best described as a union of their children. The “Phenylpropanoids and polyketides” category currently has 34 direct children and a total of 273 descendant categories, including Flavonoids, among others. Describing a flavonoid compound as a phenylpropanoid instead of a chromone (a term that can legitimately be used to describe flavonoids) is, from a biochemist’s point of view, more precise and accurate.

### Mapping of other classification schema and vocabularies to ClassyFire’s taxonomy

As noted before, there are a number of well-known, online chemical databases that have developed their own, manually annotated chemical taxonomy and/or ontology. For instance, the ChEBI ontology [15] provides a sub-ontology for chemical roles, in addition to the structure-based sub-ontology. LIPID MAPS [13] focuses on lipids and lipid-like molecules, and groups them according to their biosynthetic origin. MeSH is a thesaurus consisting of >50,000 terms, about 1/3 of which cover chemical entities or classes thereof. In developing the ChemOnt taxonomy, which is used by ClassyFire, we aimed at creating a consensus chemical taxonomy partly inspired by these approaches. In that regard, ChemOnt was mapped to three other widely used chemical hierarchies or taxonomies (ChEBI, LIPID MAPS and MeSH). This was done by assigning one or more synonyms to each ChemOnt category, and specifying the corresponding level or scope of term similarity. For any ChemOnt term, a synonym can have the identical meaning (exact scope), a more specific meaning (narrow scope), or a less specific meaning (broad scope). In some cases, the synonym can have slightly different meaning, so that it cannot be assigned any of the three aforementioned scope categories. In this case, it is simply called a related synonym.

In a joint effort with the ChEBI development team, an ontology look-up table was created to map ClassyFire’s (and ChemOnt’s) taxonomy to the ChEBI sub-ontology of chemical entities. When applicable, an exact CHEBI synonym was assigned to the ChemOnt term. Otherwise, either one or more broad synonyms, preferably those mapped to its parent, were assigned. In some cases, narrow CHEBI synonyms were also assigned. It is worth mentioning that in the case of ChEBI, due to certain philosophical discrepancies, some terms may appear to be exact synonyms for a given ChemOnt category, but actually have a different meaning. For instance, ChEBI makes a clear distinction between “carboxylic acid” and “carboxylic acid anion”, while ChemOnt does not. Therefore, the ChEBI term “carboxylic acid” is a narrow synonym of ChemOnt’s “carboxylic acids”. A total of 6014 category mappings were created, with an average of 1.24 ChEBI synonyms per category. Each ClassyFire category has one or more mapped ChEBI terms. This effort highlighted a number of similarities, differences, and suggested some improvements (e.g.: categories to be added) for both systems. Using this training information, ClassyFire has been modified and used to annotate >43,000 small molecules from the ChEBI database. A comprehensive annotation of the ChEBI database (release 126) is provided as a supplementary document (Additional file 3), and can also be downloaded from the ClassyFire website. To date, these results have been used by the ChEBI development team to annotate more than 10,000 compounds present in the ChEBI database. In lipid biology, the LIPID MAPS consortium provides the standard chemical ontology for lipids [13]. As a result we designed the lipid subset in ChemOnt to align closely with the LIPID MAPS classification scheme. A total of 789 ClassyFire categories were mapped to one of 307 LIPID MAPS terms each. As a result, a combination of ClassyFire and LIPID MAPS ontologies was used to classify ~35,000 small metabolites, which can be accessed from the LIPID MAPS Lipidomics Gateway [31], a resource sponsored by the National Institute of General Medical Sciences [37] and the Common Fund of the National Institutes of Health [38]. As a result of this mapping, several more category assignments were added to complement the LIPID MAPS classifications. ClassyFire has also been manually mapped, although only partially, to the MeSH thesaurus, which is used in the PubChem database. So far, 844 ClassyFire categories have been mapped to at least one corresponding MeSH term, accounting for a total of 945 mappings to the MeSH thesaurus. This MeSH mapping will likely continue for another year or two.

A considerable proportion of the structures available in databases, such as PubChem, correspond to chemical mixtures. For instance, some drugs or pesticides are synthesized as mixtures of several organic compounds. ClassyFire has been programmed to classify such mixtures. The underlying algorithm allows it to assign classes while considering the organic moieties separately, and also as a whole. For instance, a mixture of an organic compound and a chlorine anion (inorganic) will be assigned the category of organic chlorine salts, among others, but not the category of inorganic compounds.

## The classification process

**Fig. 4:**
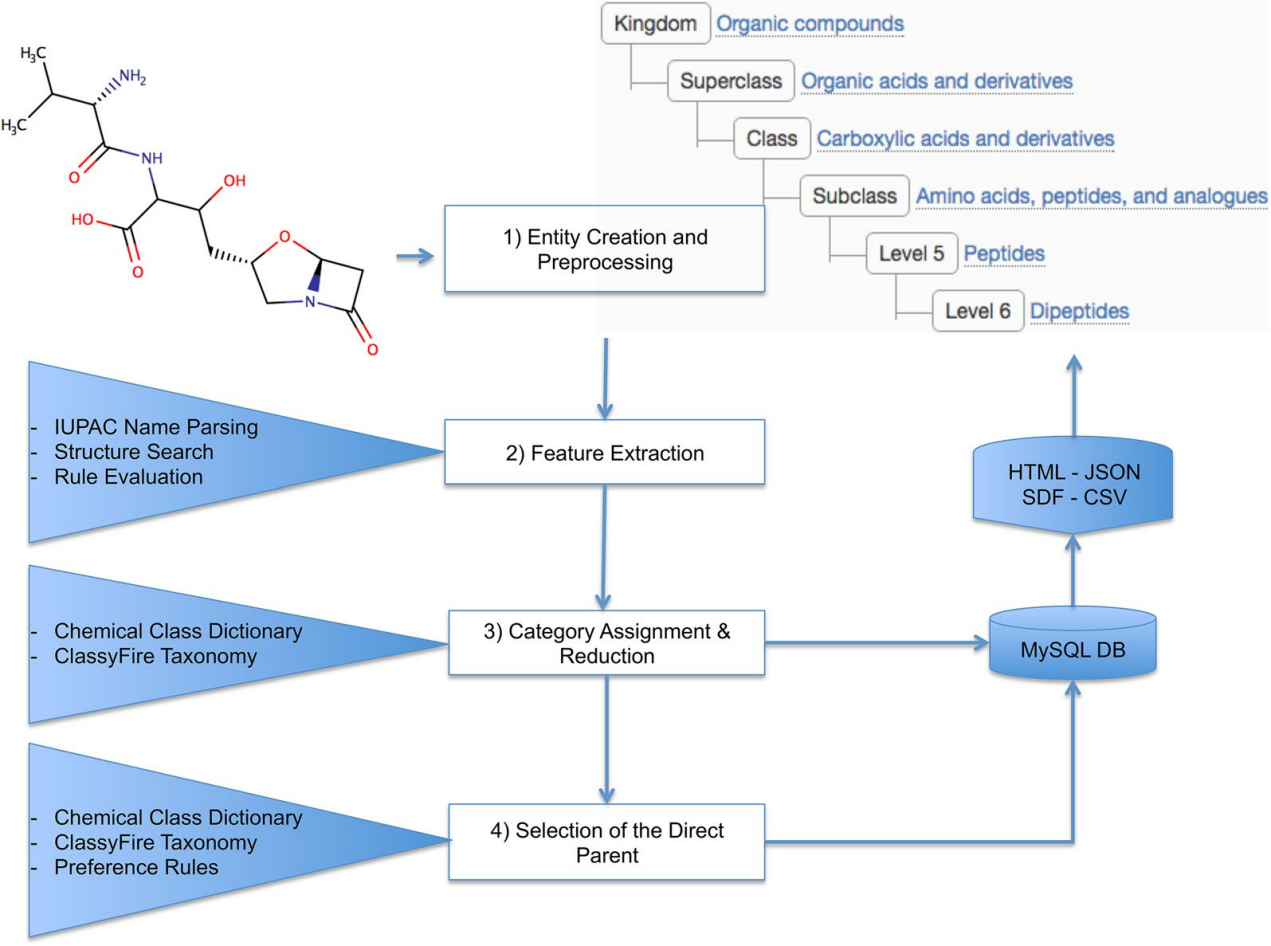
Workflow of the chemical classification

As illustrated in Fig. 4, the ClassyFire classification process involves four steps: (1) Creation and Preprocessing of the Chemical Entity; (2) Feature Extraction; (3) Rule-based Category Assignment and Category Reduction; and (4) Selection of the Direct Parent. These are described in more detail below:

### Step 1—Creation and preprocessing of the chemical entity

**Fig. 5:**
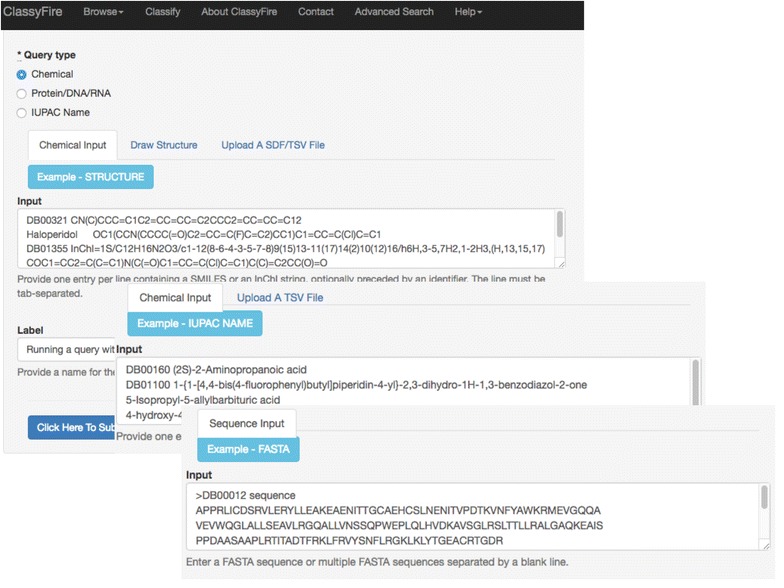
Different types of input accepted by ClassyFire

This step involves the creation of one or more chemical entity objects (which are stored in a database), and the calculation of physico-chemical as well as structural properties. Most of these features, such as the number of (aromatic, aliphatic) rings, are used for classification. Others, such as the mass, are used for text-based search (See Use Cases, below). The calculation of physico-chemical properties is performed using ChemAxon’s JChem API (version 15.5.25.0). ClassyFire accepts different types of chemical input: SMILES, SDF, InChI, IUPAC name, and FASTA sequence files. The different types of chemical input are illustrated in Fig. 5. SMILES, SDF, and InChI strings are common structural representation formats for chemical entities, which can be directly used for structure search operations or the generation of physico-chemical properties. In contrast, each IUPAC name is converted to the corresponding structure using the OPSIN library [39], before any chemical object is created and subsequently preprocessed. If the chemical (protein, DNA or RNA molecule) input is submitted in FASTA format, every sequence is either identified as a nucleotide or peptide sequence type. This step is important, as the interpretation of one-letter sequences will vary depending on the sequence type. The ClassyFire web server also allows users to submit their query through the MarvinSketch Chemical Drawing Applet, which permits users to import or draw a chemical structure, which is then exported as a SMILES string.

### Step 2—Feature extraction

The second step in the ClassyFire program involves the generation of structural features based on a combination of superstructure-search operations and various property calculations. ClassyFire combines several methods for structural pattern detection. Most features are detected through superstructure search, which is performed on its library of over 9000 manually designed SMARTS patterns and Markush structures. Each of the terms was validated through iterations of test and improvements (if necessary) over small sets of compounds. The library is integrated into ChemAxon’s JChem Base. ChemAxon’s Marvin 5.11.5 package was used to generate these patterns, ranging from small functional groups (e.g. the carbamoyl group) to complex skeletons (e.g. the (3′–>5′)-cyclic dinucleotide bis(phosphoromonothioate) pattern). Prior to being imported into the database, each structure pattern was subjected to a set of standardization operations, including normalization and aromatization. Each query compound is subjected to the same operations before the superstructure search. This allows the program to deal with differences in charges, valences and aromatic configuration.

Another feature detection method used in ClassyFire involves combining features with the use of logical connectives, and cardinality restrictions. Every structural feature defined by a logical expression is evaluated in order to assign that feature to the query compound. As an example, ClassyFire can detect specific features for an inorganic compound based on its elemental content, and the list of oxyanions it contains (if any). These features are described by rules embedded in a ClassyFire module that specifically handles inorganic compounds. In some cases, the use of structure patterns, chemical formulae or physicochemical properties is not sufficient to generate a feature. For instance, the category known as leukotrienes describes derivatives of arachidonic acid, containing three hydroxyl groups as well as four double bonds, exactly three of which are conjugated. The position of the three conjugated bonds as well as the relative position of the non-conjugated bond can vary, yielding a large number of combinations. Therefore, a superstructure search might not return a hit. In order to classify leukotrienes, ClassyFire makes use of standard IUPAC nomenclature in addition to a structure search to check whether these constraints are fulfilled. This approach is illustrated in the Additional file 2: Figure S3. The IUPAC name of any query chemical entity is generated by ChemAxon’s Structure-to-Name Conversion engine provided by the JChem API. IUPAC names can give valuable information about the parent of a given compound, as well as the positioning, number, and name of substituents relative to that parent. We developed a module, which uses a set of ~200 regular expressions and rules in order to accurately detect structural features given a query compound by parsing IUPAC names.

### Step 3—Rule-based category assignment and category reduction

After a list of structural features has been generated, each feature is then mapped to its corresponding category or node in the taxonomy. A manually compiled dictionary, which provides the weight and category for each feature, was used for the rule-based category assignment. After the category assignment is complete, a non-redundant list of chemical categories is constructed. This is done by iteratively reducing the set of chemical categories. For every pair of chemical categories, if there is a parent–child relationship (e.g. dioxanes [parent] and 1,2-dioxanes [child]), only the child node is retained (1,2-dioxanes).

### Step 4—Selection of the direct parent

The direct parent is the category defined by the largest structural feature that describes the compound. It is selected from the non-redundant list of categories obtained in the previous step. If two or more structural features have the largest weight, the direct parent is selected following a procedure that takes into account the number of cycles, heterocycles, ring atoms, ring heteroatoms, halogen atoms, fused rings, and the total number of heteroatoms, which are encoded in each node’s structural key. In some cases, the largest feature might be less descriptive or less relevant than another feature. For example, the glycoside moiety of a flavonoid glycoside can be much larger than the flavonoid moiety. However, the term “flavonoid glycoside” is more informative than the term “glycoside”, as it describes the presence of both a saccharide unit and a flavonoid, glycosidically linked to one another. In this case, an exception is made and the term “flavonoid glycoside” is selected over “glycoside”. A small (but not exhaustive) set of such exceptions has been manually compiled.

**Fig. 6:**
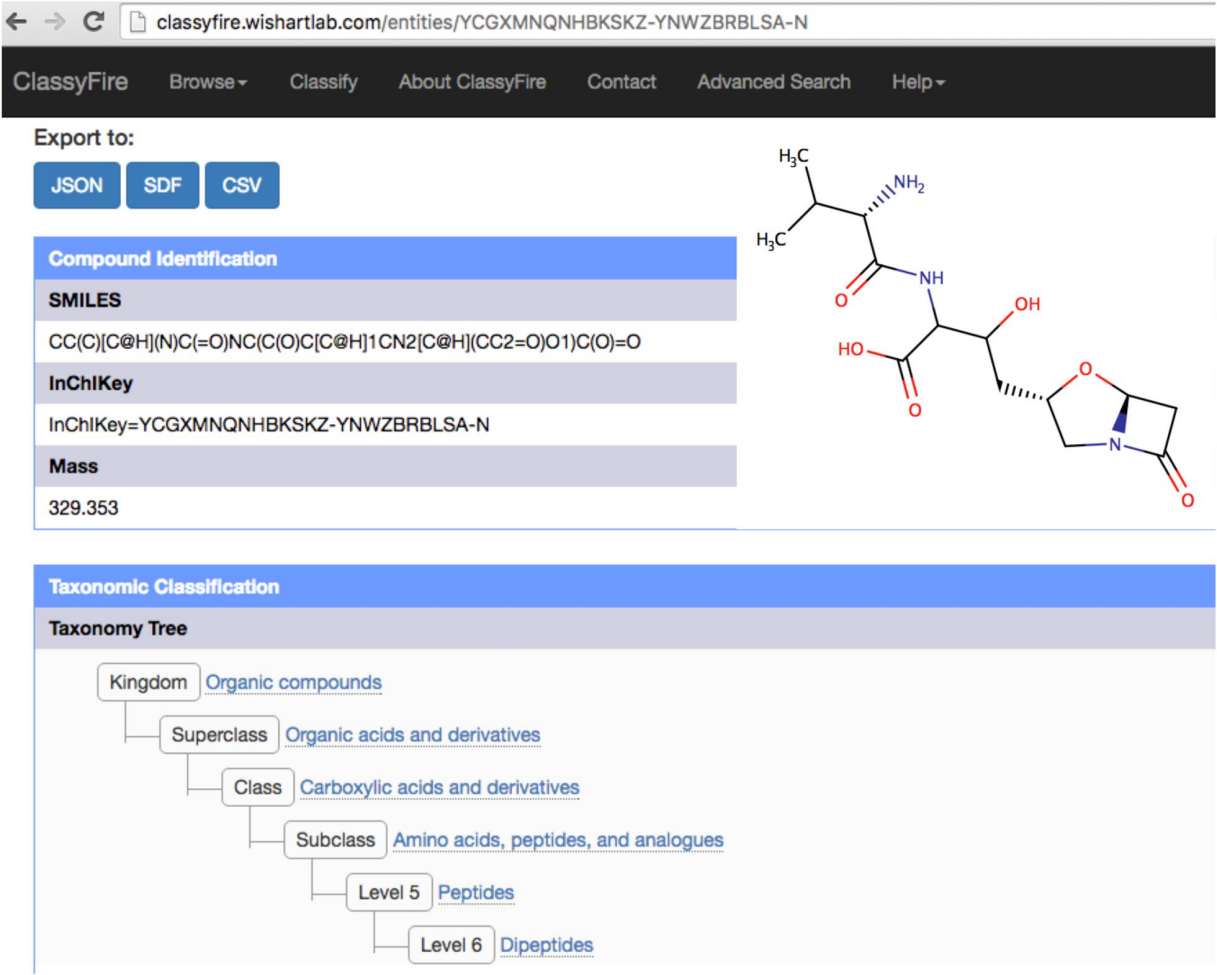
Classification results for the molecule Valclavam (CID126919) on the ClassyFire website. The structural representations, and the taxonomic tree are illustrated. The classification result can be downloaded in different formats

**Fig. 7:**
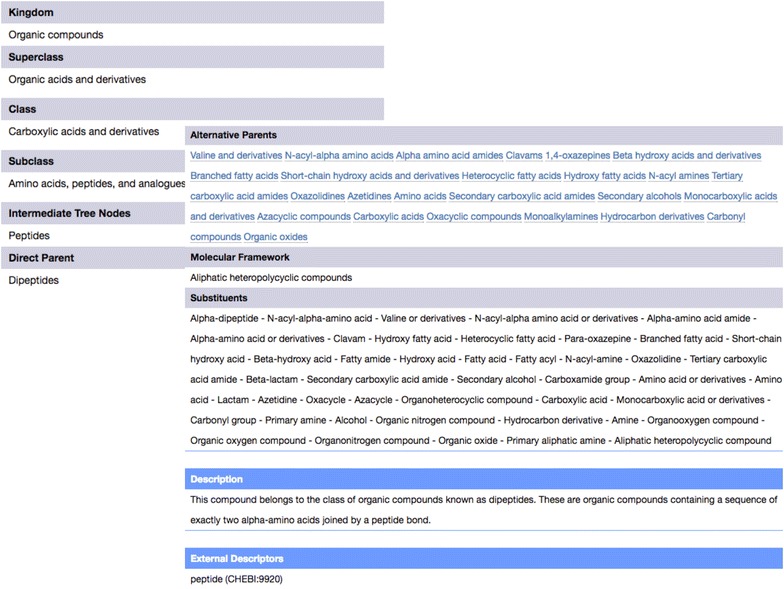
Classification results for the molecule Valclavam (CID126919) on the ClassyFire website. A detailed listing of the structural features of the molecule is provided, along with a structure-based text description

The entire ClassyFire program has been converted to a web-based resource. It is a RESTful web application located at http://classyfire.wishartlab.com. It allows users to submit one or more query molecules in SMILES, SDF, or InChI format, IUPAC name, or 1-letter amino acid and nucleic acid (FASTA) notation. The query structure(s) can be entered as text, uploaded, or drawn using the MarvinSketch applet. It is recommended that all query structures be represented in their chiral or isomeric form, to ensure a more precise classification. This is because different ClassyFire categories can be represented by stereoisomers of the same skeleton. Some examples include 3-alpha-hydroxysteroids (CHEMONTID:0003232) and 3-beta-hydroxysteroids (CHEMONTID:0003233), which are all sub-categories of 3-hydroxysteroids (CHEMONTID:0003027). When represented with an isomeric structure string for instance, a compound, such as androsterone, can be classified as a 3-alpha-hydroxysteroid. However, if it is represented with a canonical structure, it would only be classified as a 3-hydroxysteroid, which is less precise. Upon submission, the queries are processed by the ClassyFire classification tool, then entities or sequences are classified, and the results are then further processed, formatted and shown on a HTML output page (Figs. 6, 7). Classification results can also be downloaded in a JSON [40], SDF [41], or CSV [42] format. In addition to providing standard chemical classification data, ClassyFire also returns a list of chemical substituents, which are structural features (functional groups, substructures or motifs) contained within the molecule. For many compounds ClassyFire also provides a secondary attribute called the “Molecular Framework”. The Molecular Framework gives an overall description of the compound in terms of aliphaticity/aromaticity and number of cycles. For instance, benzene is described as an aromatic homomonocyclic compound while butanol is described as an aliphatic acyclic compound. The Molecular Framework attribute does not apply to mixtures of organic compounds. In addition to providing an automated chemical classification service, the ClassyFire web server also provides a number of powerful text-based search options, which are described later.

## Training and evaluation

Training and evaluation of the ClassyFire program was performed throughout the development of the program, using data sets from several well known databases, containing thousands of drugs [30], lipids [13, 32], food compounds [43], toxins, environmental pollutants, as well as other organic and inorganic compounds. Progressively larger and more diverse sets of manually classified chemicals (from 100+ compounds to more than 6000 compounds) were manually compared and evaluated against the computed ClassyFire classifications to ensure that the program properly classified new compounds or compounds not previously seen in its training cycles. The manual classifications were generated according to the definitions found in the Chemical Classification Dictionary. Moreover, classifications of the various compounds were collected from the literature and other resources that provided the same category descriptions as ClassyFire. As errors or programming bugs were identified, class definitions were iteratively refined. If missing categories were found, or if compounds were more suitably classified in new categories, these were added to the Chemical Classification Dictionary (and to the ClassyFire algorithm). The identification of new categories was aided by the classification schema provided by other databases such as LIPID MAPS [13], ChEBI [15] and DrugBank [30]. This iterative refinement process was conducted until essentially no incorrect assignment could be detected in even the largest test sets.

In addition to these manual consistency checks conducted throughout the training and development phase of the project, we also conducted an independent performance assessment of the final release version (version 2.0) of ClassyFire. A test set was built by randomly selecting 800 unique structures from DrugBank, the LIPID MAPS Lipidomics Gateway, HMDB [32], and T3DB [44]. The compounds are all included in the PubChem database. We used a panel of experts to evaluate the correctness of each category assignment based on the definition in the Chemical Classification Dictionary. When applicable, we also verified if the direct parent was included in the list of classed assigned by ChEBI or LIPID MAPS.

## Results and discussion

The classification process as described in the previous section was implemented into both a computer program and a freely accessible web server called ClassyFire, available at http://classyfire.wishartlab.com. Moreover, an open source Ruby API (https://bitbucket.org/wishartlab/classyfire_api) allows users to programmatically access the web server in order to submit queries, and retrieve classification results, as well as entity-related properties. The complete taxonomy can be downloaded from ClassyFire’s home page.

An example of ClassyFire’s classification and ontological annotation is illustrated for the antibiotic compound Valclavam. As can be seen in this figure, ClassyFire returns a taxonomic classification based on the most descriptive node in the taxonomy (Fig. 6). The direct parent “dipeptides” represents the most dominant moiety of Valclavam’s structure. However, the notion of what is most descriptive can vary from one user to another, and from one context to another. For example, a cyclic depsipeptide could be also be classified as a lactam. Because of this ambiguity, ClassyFire also displays a list of Alternative Parents (Fig. 7) providing a more detailed description of the chemical. Alternative parents are categories that describe the compound but do not have an ancestor–descendant relationship with each other or with the Direct Parent. When available, ClassyFire returns Intermediate Nodes. These are nodes are descendants of a subclass (any category with a depth of 4), but have a depth lower than the direct parent.

In addition, ClassyFire provides the Molecular Framework and a list of all identified substituents (or structural features). Furthermore, an English, text-based compound description is also provided for non-experts. The text-based description is derived from ClassyFire’s Chemical Classification Dictionary. In an effort to facilitate the integration of data from different sources, ClassyFire also contains a database of cross-references from other popular chemical databases that use different taxonomies/ontologies, such as KEGG [45], ChEBI [15], LIPID MAPS [13], and MetaCyc [46]. These cross-references and alternate-database classifications are routinely provided as ClassyFire output, when available.

To accelerate ClassyFire’s processing time, all of the chemical structures it has ever processed and all of the corresponding taxonomic/ontological outputs it has ever produced are stored in a local MySQL database. This allows the ClassyFire web sever to perform a simple lookup for those query compounds that have previously been processed (more than 70 million compounds to date). Therefore, for previously analyzed compounds the ClassyFire web server takes <50 ms to return an answer. For completely novel compounds, the ClassyFire web server takes an average of 540 ms to classify a structure.

## Evaluation of ClassyFire’s classification results

**Fig. 8:**
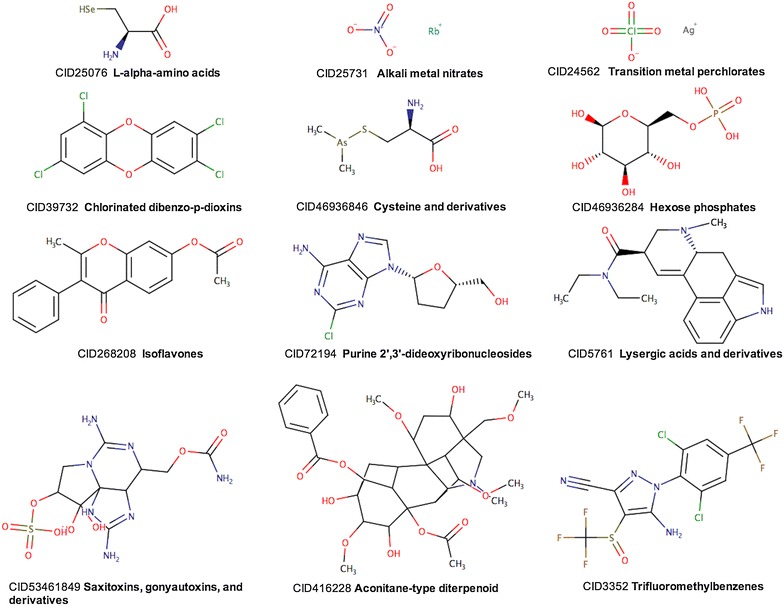
Examples of class assignments by ClassyFire for 12 compounds from the test set

After the iterative development, testing and manual evaluation of ClassyFire over several data sets consisting of >30,000 compounds from very diverse chemical categories, ClassyFire was formally tested on a set of 800 compounds not used during ClassyFire’s training phase. The compounds among which, drugs, food compounds, synthetic compounds, and biologically relevant metabolites, were selected from PubChem (Additional file 4: Sheet 1). The classification process took 249.9 s on a computer with 4 CPU CentOS nodes, with 3.6 GB of RAM, running with a maximum of 16 threads. The results were then manually reviewed by a panel of seven chemistry experts from three different countries (Additional file 4: Sheet 2). A total of 21,102 category assignments were made, for an average of 26.38 assignments per compound. On this specific test set, ClassyFire assigned a total of 1308 distinct Categories. Figure 8 illustrates some examples of the category assignments. The goal was to evaluate how exact the computational rules were able to reflect the text-based descriptions, which themselves are traditionally used to classify compounds. Based on these textual descriptions, as well as the assignments from the literature and scientific databases, each compound’s annotation was reviewed to identify possibly missing or wrong assignments.

**Fig. 9:**
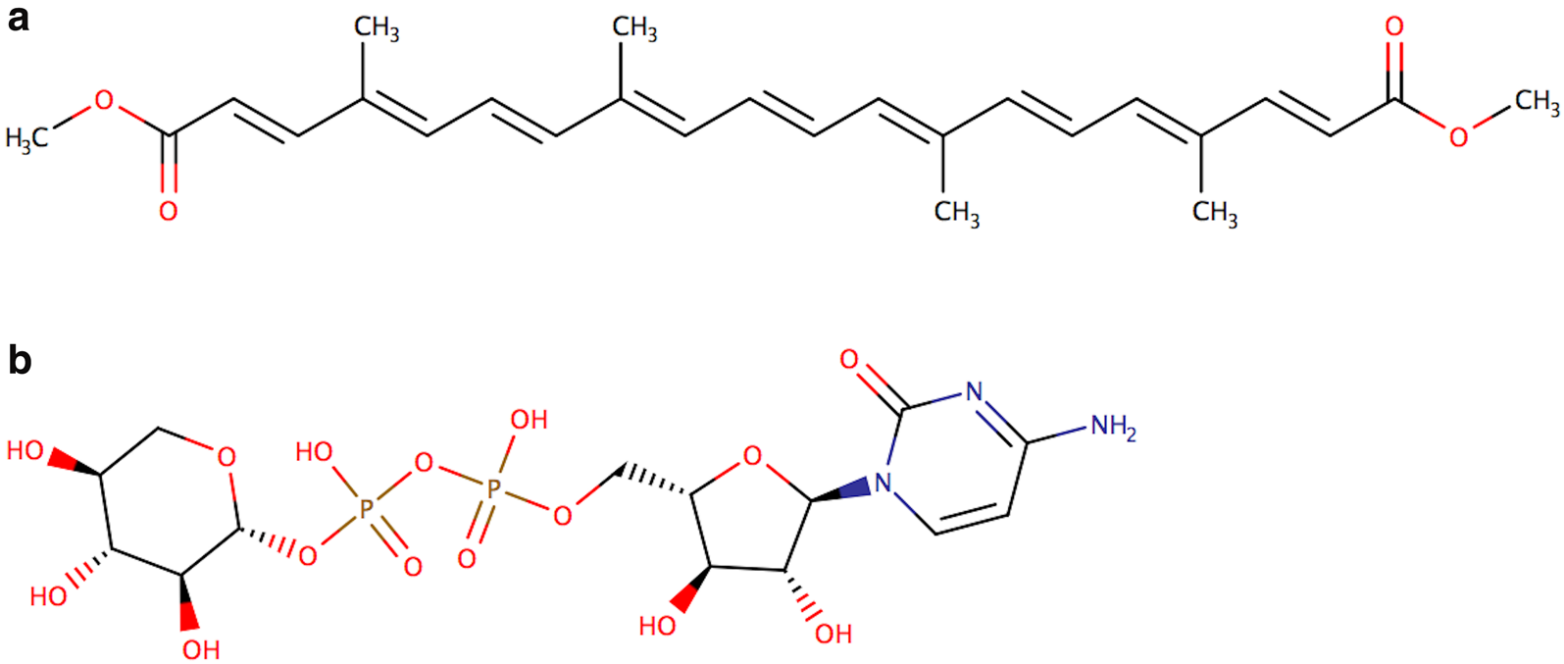
Examples of conflicting and missing class assignments. **a** Structure of Bixin dimethyl ester (CID14413719). **b** Structure of cytidine-5′-Diphospho-Beta-d-Xylose (CID 46936568)

In this test, a total of 17 false positives (out of 21,102 assignments) were detected. An example is the misclassification of bixin dimethyl ester (CID14413719) as an acyclic diterpene. From a structural point of view, this compound contains a chain of four consecutive isoprene units, which is characteristic of diterpenes (Fig. 9a). However, bixin dimethyl ester is classified in both the LIPID MAPS and the ChEBI database as a C40 isoprenoid (tetraterpene). More precisely, bixin dimethyl ester belongs to the category of compounds known as apo-carotenoids, which arise from the oxidative cleavage of carotenoids. Thus, bixin dimethyl ester, which is a product of lycopene metabolism, is classified as a tetraterpene according to its biosynthetic origin. Based on its structure, one could argue that bixin dimethyl ester should be classified as a diterpene; but based on its biology, it should be classified as a tetraterpene derivative or as an apo-carotenoid diterpenoid (CHEBI:53186). Given that ClassyFire is designed to classify compounds on a structural basis rather than a biological or biosynthetic basis, this kind of “misclassification” is completely understandable and is arguably not a misclassification. In this test set we also detected 13 missing assignments (false negatives). An example of a compound missing an assignment is the experimental drug cytidine-5′-diphospho-beta-delta-xylose (CID46936568), which was only classified as a pyrimidine ribonucleoside diphosphate but not classified as a purine nucleotide sugar (Fig. 9b).

To evaluate ClassyFire’s overall performance, each category was assigned a normalized weight based on its number of occurrences among the 800 chemical entities. This way, incorrect or missing assignments of the more populated categories (e.g. those at a higher level of the taxonomic hierarchy) would be penalized more compared to less populated categories (i.e. those at a lower level of the hierarchy). Each category was assigned to an average of 2.6 compounds. ClassyFire obtained score of 7067.04, or 99.97% of a maximum score of 7067.24. On average, ClassyFire was able to reproduce the text-based description with a precision of 99.8% and a recall of 99.9%.

### Comparing automated and manual annotations

The primary motivation behind automated chemical classification is to provide a comprehensive, accurate and fast chemical annotation in order to alleviate the cost and potential errors of manual classification. While ClassyFire is many times faster than manual classification methods we also wanted to assess its accuracy and completeness compared to manual classifications. We therefore conducted a detailed comparison of ClassyFire’s results from 20 compounds, randomly selected from the test set described above, with their manually curated annotation from the ChEBI database. The 126th ChEBI release from April 1st 2015 was used for this comparison. We did not use a more recent version of ChEBI since ClassyFire has actually been used over the past year to guide the manual annotation process for the ChEBI database. In order to provide the complete ChEBI annotation, a script was used to infer a list of ancestors for each of the 20 compounds based on the selected ChEBI release. Each compound was assigned an average of nearly 33 ChEBI classes (Additional file 5: Sheet 1). ClassyFire, on the other hand, returned an average of ~31 categories per compound. The ontology lookup table described in the Methods section of this paper was used to map categories returned by ClassyFire to the ChEBI classes. This mapping returned an average of 27 terms, or approximately 6 terms less than that originally provided by ChEBI.

This discrepancy can be explained by several factors. First, the idea behind the term mapping was to assign each ChemOnt category to an equivalent ChEBI term or, if not applicable, the closest ChEBI classes that do not have a parent–child relationship to each other. Thus, the category “Primary amines” (CHEMONTID:0002450) has been mapped only to the equivalent ChEBI term “primary amine” (CHEBI: 32877), and not its parent. Additionally, the two hierarchies are built differently. While ChemOnt is built as a tree, where each node has no more than one parent, a ChEBI term can have several parents. For the purpose of our comparison, we complemented the list of the predicted ChEBI terms with their inferred parents (Additional file 5: Sheet 2). When the extended list is considered, each compound in the set was assigned to a total number of nearly 45 predicted ChEBI terms. Of those, an average of nearly 14 terms were missing from the manual ChEBI annotation. These could be added to ChEBI in order provide a more complete and consistent annotation. From the 33 terms provided by ChEBI, ClassyFire was unable to return an average of more than 2 terms per compound. This could either suggest that more terms should be added to the ChemOnt hierarchy, or the lookup table could be improved. In some cases, the term used is based on both a structural and a functional classification. An example is the term beta-lactam antibiotic (CHEBI:27933) for Oxacillin (CID 6196). Because ChemOnt is strictly structure-based, these terms do not apply. Overall, ClassyFire was able to reproduce ~94% of the ChEBI annotations, but also to suggest new terms that could accurately increase the number of annotations by another 43.6%.

The approach presented in this work makes use of diverse cheminformatic technologies to precisely detect structural features and classify chemical entities. The ClassyFire classification algorithm helps to (partially) overcome many of the limitations of previously developed automated chemical classification tools [24, 26, 27]. For instance, several rules were developed to classify inorganic compounds, and organic metal compounds, which are not comprehensively covered by any current ontology. Most categories e.g., benzodiazepines, can be accurately described by one or more structural patterns. Others, such as alkaloids and derivatives, can only be defined as a disjunction of several subcategories. Furthermore, ClassyFire makes used of IUPAC names to identify certain patterns that might not be retrieved by a standard structure search, due to different substitution or dehydrogenation patterns. For example, we described a method to classify leukotrienes based on IUPAC names, given that there is no single structural backbone that could sufficiently and accurately describe each of these compounds.

### Limitations

Despite the many capabilities that ClassyFire offers, and the different methods used to circumvent some of the formalization problems mentioned so far, certain limitations in ClassyFire still remain. For instance, ClassyFire’s reliance on IUPAC names as a classification feature continues to cause some problems, particularly for compounds such as leukotrienes. This is because the classification of leukotrienes is also partly based on their biosynthetic origin. Certain, leukotriene derivatives that are oxidized or reduced at one double-bond position are still classified as leukotrienes, even though they might no longer have the three conjugated double bonds or the fourth double bond. An example is 10,11-dihydro-12-oxo-LTB4 (LMFA03020041) found in the LIPID MAPS database. Improvements could be made by taking a closer look at such compounds to find more common structural patterns. Currently, these leukotrienes would be classified as hydroxyeicosadienoic, hydroxyeicosatrienoic, or other eicosapolyenoic acids, depending on the number of carbon–carbon double bonds. Additionally, IUPAC names can become very difficult to exploit for certain complex structures, such as large fused ring systems. Another limitation with ClassyFire lies on its heavy dependence on predefined chemical patterns that use imperfect structure representation formats. Because ClassyFire inherits some of the limitations found with standard chemical structural representations (i.e. SMILES, SMARTS, Markush), the classification accuracy for certain kinds of “sandwich compounds” (e.g. metallocene) and alloys (e.g. chromium alloys) is not as good as it could be.

In order to circumvent the aforementioned limitations, and for the sake of developing a standard taxonomy, ClassyFire and ChemOnt could benefit from the involvement of the International Union of Pure and Applied Chemistry (IUPAC), and other chemical standardization or data reporting bodies. These groups could help to propose newer/better classifications and provide the long-term continuity that would, in turn, help to achieve a more sustainable and more consensual approach to chemical classification. Currently, the ClassyFire code is compatible only with the commercial ChemAxon JChem package. In order to ensure the sustainability of ClassyFire, we are committed to rapidly (by December 2016) making ClassyFire a completely open source project that could benefit from contributions from the global cheminformatics community. ClassyFire’s continued maintenance and further development will be achieved under the joint supervision of The Metabolomics Innovation Center (TMIC), the National Institute of Health (NIH), the European Bioinformatics Institute (EBI), as well as IUPAC. We believe that this would facilitate the involvement and more widespread adoption of ClassyFire and ChemOnt, by the scientific community.

## Use cases

As mentioned earlier, the benefits and applications of a comprehensive chemical classification schema and well-defined chemical ontology system are multifold. Chemical classification makes chemical information easy to index, easy to organize, easy to search and easy to exchange. It also makes it possible to automate chemical annotations, to perform complex chemical searches, to rapidly identify compounds for compound-specific predictions, and to decipher patterns that underlie key biomolecular interactions. To illustrate this, we provide some example use cases showing how ClassyFire’s chemical classification has been used to help solve some common cheminformatics tasks.

### Example 1: Classification of the PubChem database

PubChem [19] is a freely available chemical database maintained by the National Centre for Biotechnology Information. It stores chemical, physicochemical and biological information for more than 91 million chemical entities as of June 2016, making it the largest, open-access chemical database in the world. However, as large as PubChem is, only 0.12% of the compounds in the database have ever been assigned to a chemical class or given a Medical Subject Heading (MeSH) classification. MeSH is a manually maintained, controlled vocabulary produced by the National Library of Medicine. It is used for indexing, cataloging, and searching for biomedical and health-related documents, including all abstracts and papers listed in PubMed [47]. Over the past 40 years, MeSH classifications have been assigned manually for just 115,000 compounds in PubMed, yet there are 60 millions compounds listed in PubChem. Given that the number of documents listed in PubMed is rapidly increasing, a manual assignment of the MeSH classes will become increasingly difficult. Moreover, it would be impossible to manually annotate all 60 million compounds in PubChem using the standard MeSH methodology. Therefore, we decided to automatically annotate and classify all of PubChem (and all PubMed chemicals) using ClassyFire. The structure-based classification of PubChem compounds was performed through parallel computing on 22 CentOS quad-core CPUs, with 3.6 GB of RAM each. The operation was completed in 424 h for an average of 550 ms (ms) per compound. The classification results have been submitted to the PubChem development group. This group is actively working to display ClassyFire’s classification of all the PubChem compounds, thereby allowing users to view, query and access compounds based on their ChemOnt classification. This should be completed by late 2016. With PubChem fully classified, the indexing of PubMed documents will now be much easier. Combining structure-based annotations with biological data could also assist scientists in various projects, such as ontology-based chemical enrichment analysis [17]. Moreover, through ClassyFire, it is now possible to perform a variety of fast data searches and retrievals of PubChem data, as outlined below.

### Example 2: Fast searching and data retrieval

Chemical databases can typically be queried via physico-chemical parameters (e.g. mass) while others can be searched for the presence of functional groups (e.g. a ketone or carboxylic acid), among other properties. However, querying a chemical database with both substituent constraints and mass constraints is very difficult. For large databases, this would require one to perform structure-based searches over millions of compounds, which can take several minutes, even when the compounds are fully indexed. Moreover, certain structural constraints cannot be expressed using conventional structure-handling formats, such as SMARTS. Additionally, conventional substructure or structure-based searches do not allow one to search for chemicals belonging to categories such as “Alkanes” or “Alkaloids and derivatives”. Having a chemical database annotated with substituent or chemical classification information can make these kinds of substituent and mass constraint searches very fast and easy. ClassyFire supports exactly this type of flexible search as it allows users to select compounds by defining a set of conditions based on various parameters such as, the chemical category, the mass, the number of rings, etc. These types of search combinations are very common in fields such as mass spectrometry, where compounds must be identified based on physico-chemical properties and relatively vague information about their putative substituents. ClassyFire’s text search operations are supported by Elastic Search [48], an open source search and analytics engine. As a result, compounds can be selected from over 77 millions compounds stored in the ClassyFire database (as of June 2016) based on the ChemOnt terminology. Additionally, when needed, the results can be filtered based on physico-chemical properties. An illustration of how such a search can be conducted is provided in the Additional file 6: Figure S4, where ClassyFire returned a list of “Alkaloids containing more than one ring or, and having a mass lower than 700 daltons”. The operation returned 30,392 hits through its text-based search in 509 ms. The results of the text-based search could be used to identify unknown structures obtained from biological samples. They could also be used to explore and cluster sets of small-molecules isolated from metabolomics or natural product extraction experiments.

### Example 3: Automated chemical annotation

A growing number of chemical databases are being developed wherein detailed descriptions of individual chemicals are required. Examples include MetaCyc [46], ChEBI [15], DrugBank [30], T3DB, ECMDB [49] and FooDB [43]. In many cases these descriptions must be manually composed and edited by experts and annotators. For well-known chemicals writing a comprehensive description is trivial. However, for lesser-known chemicals or chemicals where very little literature is available, the preparation of an even a short textual description of 20–30 words can take hours of library sleuthing and reading. Because ClassyFire has a comprehensive Chemical Classification Dictionary consisting of thousands of 20–50 word textual descriptions for different compound classes, it is possible to use this Dictionary to automatically describe or annotate obscure or little-known compounds. In particular, ClassyFire was used to generate over 13,100 meaningful, 20–50 word descriptions for compounds in, ranging from drugs to poisons, for which no literature data was available. These precise, but automatically generated compound descriptions are now available in the HMDB, ECMDB, T3DB, FooDB, and YMDB [50].

## Conclusion

In this paper, we have described a comprehensive, computable chemical taxonomy along with a structure-based ontology that permits the fully automated classification of most of the world’s known chemicals. In particular we have described: (1) an well-defined, hierarchical classification structure consisting of up to 11 taxonomic levels; (2) a freely available Chemical Classification Dictionary (or ontology) consisting of >4800 carefully identified and precisely described chemical classification terms, with over 9000 synonyms; (3) a set of >9000 objective rules, patterns and criteria for classifying compounds on the basis of their structure; and (4) a computer program and a freely available web server (called ClassyFire) that performs rapid, accurate, automated rule-based taxonomic classification of chemical compounds. To our knowledge, this is the first freely available system that is capable of automatically, accurately and comprehensively organizing most of the world’s known chemical entities into structural classes, at the scale presented.

The flexibility of ClassyFire’s source code and ChemOnt’s chemo-taxonomic definitions, along with their open accessibility should allow ClassyFire and ChemOnt to easily evolve to fit with the ever-changing views of chemistry and with the increasing number of newly discovered scaffolds of natural and synthetic chemicals. In addition to developing an extensive taxonomy of organic compounds, we have also developed a comprehensive taxonomy for inorganic compounds consisting of 674 categories based on molecular formulas and atom types. We believe this is the first significant attempt to design a comprehensive computable chemical taxonomy for inorganic compounds.

ClassyFire’s performance shows that the classification of chemical compounds can be accurately computed in a rapid, dataset-independent manner by relying solely on structural properties. Our data suggests that most chemical classes can be represented by one or more structural patterns. In certain cases, however, compounds from a given chemical category undergo reactions (e.g. loss of oxygen, substitutions) that might not match the constraints described in a category description. Some approaches to provide accurate descriptions in these scenarios would be to add more patterns, update position-specific constraints, and/or develop some heuristics for a more accurate classification. For instance, creating more rules for IUPAC name parsing could help to assign some classes more accurately. Overcoming these limitations would certainly improve the overall performance of ClassyFire.

It is important to emphasize that this taxonomic effort was not done in isolation. It has been jointly developed and tested by curators and developers some of the largest and most popular open-access chemical databases in the world, including PubChem, ChEBI, LIPID MAPS, DrugBank, HMDB and others. The ClassyFire/ChemOnt taxonomy is already being used in several of these databases and is expected to be adopted by several other chemical databases in the near future. Furthermore, the entire ClassyFire/ChemOnt taxonomy was mapped, in a joint effort, to several existing taxonomic/ontological schemes, such as the ChEBI and LIPID MAPS ontologies. As illustrated with the previous examples, applications of ClassyFire are multifold, spanning areas including drug design and metabolomics. ClassyFire has also found applications in the field of Chemical Health and Safety, where hazard assessment of small molecules, based on their structural features, has gained increasing interest recently.

ClassyFire is obviously not the final word on chemical classification or chemical taxonomies/ontologies. Given the size and complexity of the global chemical space along with the rapidly evolving needs of chemists and cheminformatics specialists, we expect that this subject (and this software) will evolve considerably over the coming years. Therefore, besides the freely available web service, we are actively working on a version of ClassyFire that has freely accessible source code and documentation. We are committed to making this resource fully open source (by December 2016). We believe this effort is an important first step towards the design of a fully computable, universally accepted chemical taxonomy and ontology.

## Additional files

**Additional file 1.** Description of the chemical taxonomy, and terms for the hierarchical classification.

**Additional file 2.** ClasssyFire’s approaches for the feature extraction.

**Additional file 6.** Comprehensive annotation of the ChEBI database (release 126).

**Additional file 3.** ClassyFire’s category assignments for a test set of 800 compounds.

**Additional file 4.** Comparison of ClassyFire annotations to ChEBI labeling (Statistics and Category assignments).

**Additional file 5.** Use cases. Text-based search on the ClassyFire web server. (A) Building the query. (B) Sparteine, one of the returned compounds.

## Abbreviations

CSV: comma-separated values
IUPAC: international union of pure and applied chemistry
JSON: JavaScript object notation
SDF: structure data file
SMILES: simplified molecular-input line-entry system (SMILES)
