# Supplementary material for: ClassyFire: automated chemical classification with a comprehensive, computable taxonomy
Source: J Cheminform. 2016 Nov 4;8:61. doi: 10.1186/s13321-016-0174-y (PMC5096306; doi:10.1186/s13321-016-0174-y)
Supplement: Supplementary file 1 — Additional file 1. Description of the chemical taxonomy, and terms for the hierarchical classification. [file 13321_2016_174_MOESM1_ESM.docx]

**Mathematical definition of ChemOnt, the ClassyFire’s taxonomy**

The presented structure-based ontology is defined as a directed hierarchy tree T (N, A) where: Each node n in N represents a specific set of chemical entities,

1. Each directed arch (n, m) ϵ A means that node m is a subset of the node n, and
2. A predicate $c_{n}\left( x \right)$for a chemical $x$is true if and only if $x$ belongs to the node n, and for each arc (n, m) ϵ A${: c}_{n}\left( x \right)=>c_{m}\left( x \right).$

In this context, a node represents a chemical class, which is a set of chemical entities sharing a common structural property (or a set of properties). If the common property is a core backbone (e.g.: a beta-lactam backbone), then a varying number of parts (including substituted groups) can be attached to it, to define a specific subclass or a unique compound. If the common feature is expressed as a logical term or a combination of connected logical terms, then a specific subclass can be defined through conjunction with another term. We use the “*is_a”* relationship to link each node to its parent. The “*is_a”* relationship is transitive. This means that for every triplet (A, B, C) of chemical categories in the tree, $A is\_a B \bigwedge B is\_a C\to A is\_a C$. Moreover, the tree is “*is_a”* complete. This means that for every node in the tree, there is a path leading to the root of the tree using the “*is_a”* relationship.

For every node representing a category of organic compounds in the ontology, the parent node was determined using the following approach:

1. Organic compounds are compounds that contain at least one carbon atom, with the exception of the following: isocyanide/cyanide and their non hydrocarbyl derivatives, thiophosgene, carbon diselenide, carbon monosulfide, carbon disulfide, carbon subsulfide, carbon monoxide, carbon dioxide, Carbon suboxide, and dicarbon monoxide. These compounds were selected from the list of inorganic carbon compounds in the literature. We note that for a number of inorganic compounds, there is not a clear line of whether to consider them as inorganic or organic. Moreover, the list of exceptions could be subjected to changed in future versions
2. The parent node must also be a descendant category of organic compounds;
3. If the node is represented by a core backbone *C*, the candidate parents are determined by removing at least one peripheral part or bridge in the backbone, resulting are a list of n parent backbones *P_1_... P_n_.* The node is assigned only the largest candidate parent. For instance, by decomposing the N-acylpiperidine backbone, piperidine is the largest backbone remaining; thus, N-acylpiperidines will be assigned the parent node
4. “Piperidines”; this differs from the scaffold tree method in that here, not all peripheral parts must be removed in one step.

If the core backbone is an unsubstituted moiety with at least three fused rings, the selected parent is the largest fused-ring system that is a substructure of the backbone. For example, “Coumarins” is the parent category of “Furanocoumarins”.

The nodes representing inorganic categories were added to the tree using the following criteria:

1. the homogeneity or heterogeneity of the compounds (metal, non-metal, mixed metal/non-metal),
2. The types of elements contained in the compounds, and
3. The presence and type of oxyanions, salt forms, as well as other functional groups.

We also added a number of classes to represent inorganic carbon compounds (such as carbides), and other groups of compounds derived from inorganic groups such as isocyanide, and azides.

| **Term** | **Definition** |
| --- | --- |
| Kingdom | First level of hierarchical classification: Organic or Inorganic. |
| Superclass | Second level of hierarchical classification. Metabolites with the same superclass share generic structural features that describe their overall composition or shape. |
| Class | Third level of hierarchical classification. Metabolites of the same class share a parent substructure. The structural similarity is generally higher at the class level compared to the superclass level. |
| Subclass | Fourth level of hierarchical classification. Metabolites of the same class share a parent substructure. The structural similarity is generally higher at the subclass level compared to the class level. |
| Intermediate nodes | Nodes that are descendants of the subclass and ascendants of the direct parent. |
| Direct parent | The category corresponding to the largest skeleton or most dominant feature of the classified compound. The direct parent could correspond to the superclass, class, subclass or any other lower level. In the latter case, the intermediate parents can be traced back using the ontology file. |
| Alternative parents | Other categories in the ontology that describe the classified compound and do not display a parent-child relationship to each other or to the direct parent. |
| Molecular framework | Provides a general description of the compound in term of aliphaticity/aromaticity, number of cycles, and the variety of atom types (homo, hetero). This is calculated only for compounds/mixtures with less than two organic moieties. |
| Substituents | Functional groups and substructures contained in the compound. Only to avoid redundancy, the substituents mapped to each category of a given ontological classification are removed from the list of substituents. |
| Description | Textual structure-based description of the compound. It gives a brief description of the main characteristics of the largest skeleton or most dominant structural feature. |
| External descriptors | Annotation of the compounds in other databases. It only shows the deepest nodes in the classification. |

Table S1: Definitions of terms used in the ontological classification.
