# Supplementary material for: ClassyFire: automated chemical classification with a comprehensive, computable taxonomy
Source: J Cheminform. 2016 Nov 4;8:61. doi: 10.1186/s13321-016-0174-y (PMC5096306; doi:10.1186/s13321-016-0174-y)
Supplement: Supplementary file 2 — Additional file 2. ClasssyFire’s approaches for the feature extraction. [file 13321_2016_174_MOESM2_ESM.docx]

***
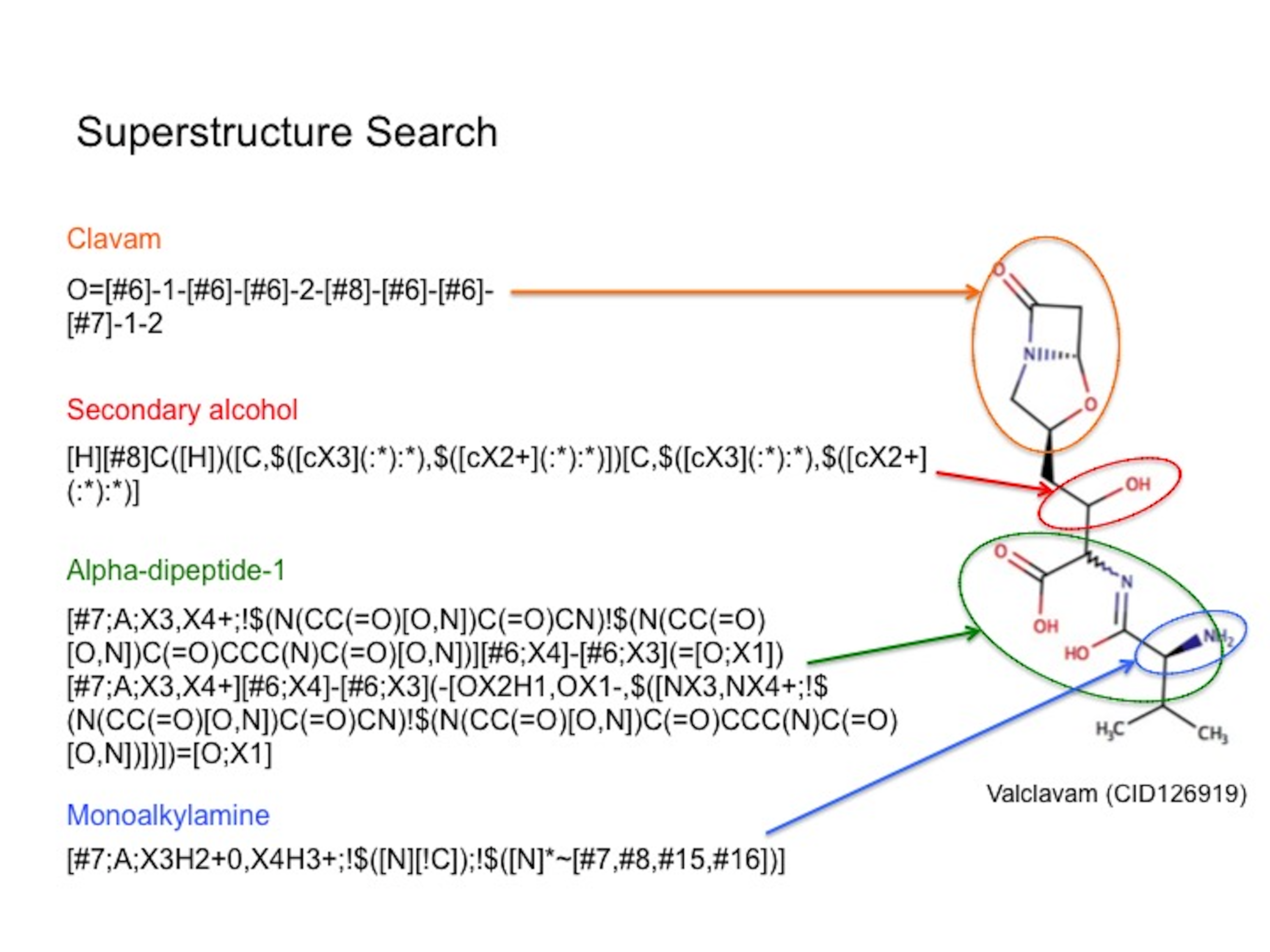
***

Figure S1: Examples of patterns found in Valclavam through superstructure search. A library of >9,000 was manually designed and tested. This library is used by ClassyFire for superstructure search operations.

***
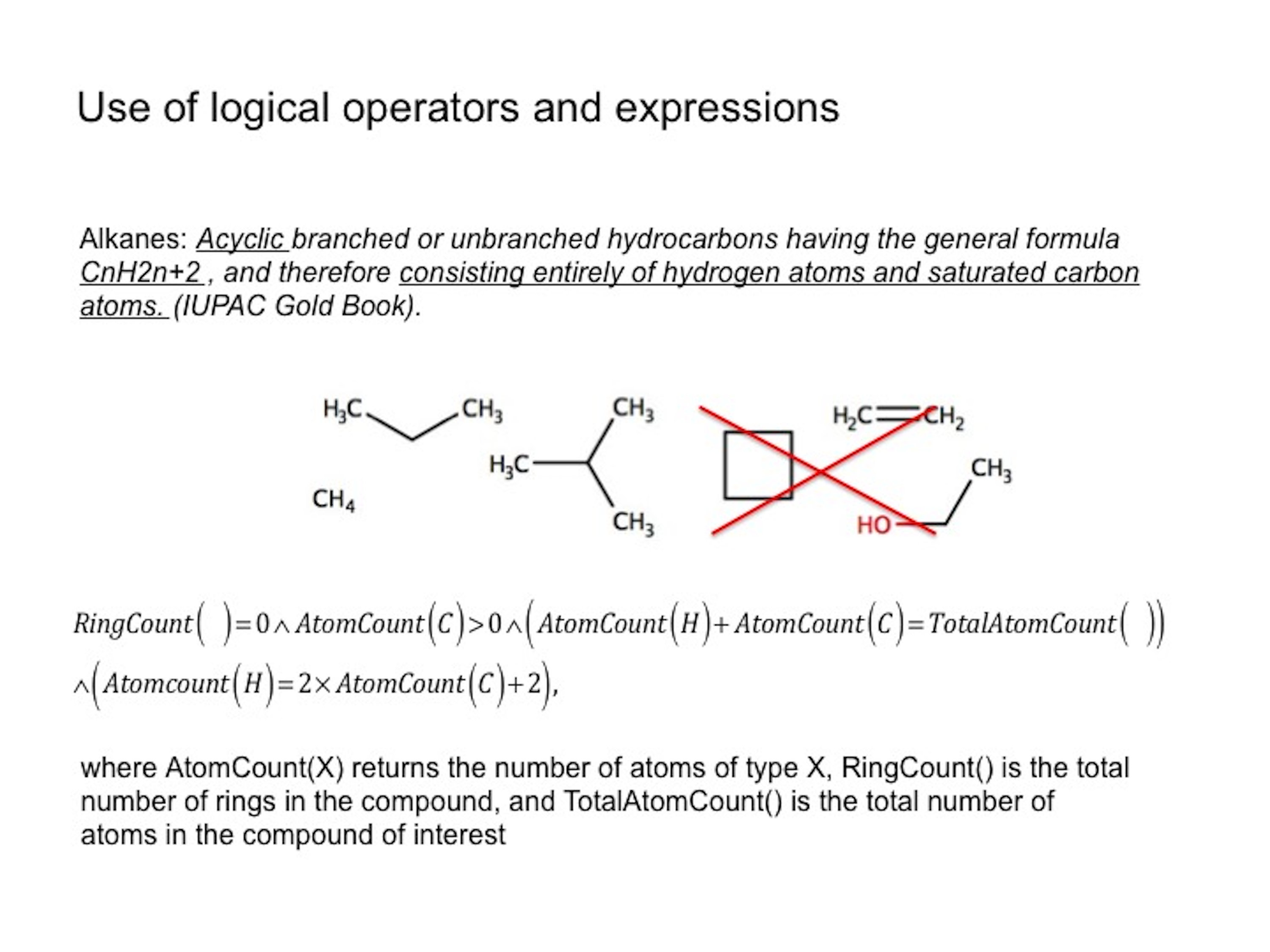
***

Figure S2: Expressing structure-based description with logical operators.

******

Figure S3A: An example approach for feature detection using the IUPAC nomenclature principles. An illustration with the example of Valclavam.

******

Figure S3B. An example approach for feature detection using the IUPAC nomenclature principles.

Leukotrienes and Lipoxins are derived from eicosatetraenoic acids. Leukotrienes differ from other eicosatetraenoic acids such as lipoxins, in that only three of the four double bonds are conjugated. The position of the double bonds can vary from one molecule to another. IUPAC name parsing will involve: 1) Parsing the IUPAC name for specific patterns, including /trihydroxyicosa[a-zA-Z]*-[0-9]+,[0-9]+,[0-9]+,[0-9]+-tetraenoic acid|[0-9]+,[0-9]+-dihydroxy-[0-9]+-oxoicosa[a-zA-Z]*-[0-9]+,[0-9]+,[0-9]+,[0-9]+-tetraenoic acid/. 2) Identifying the relative positioning of the double bonds using logic connectors. Only three of the four conjugated bonds are conjugated.
