# Supplementary material for: ClassyFire: automated chemical classification with a comprehensive, computable taxonomy
Source: J Cheminform. 2016 Nov 4;8:61. doi: 10.1186/s13321-016-0174-y (PMC5096306; doi:10.1186/s13321-016-0174-y)
Supplement: Supplementary file 6 — Additional file 5. Use cases. Text-based search on the ClassyFire web server. (A) Building the query. (B) Sparteine, one of the returned compounds. [file 13321_2016_174_MOESM6_ESM.pdf]

A)

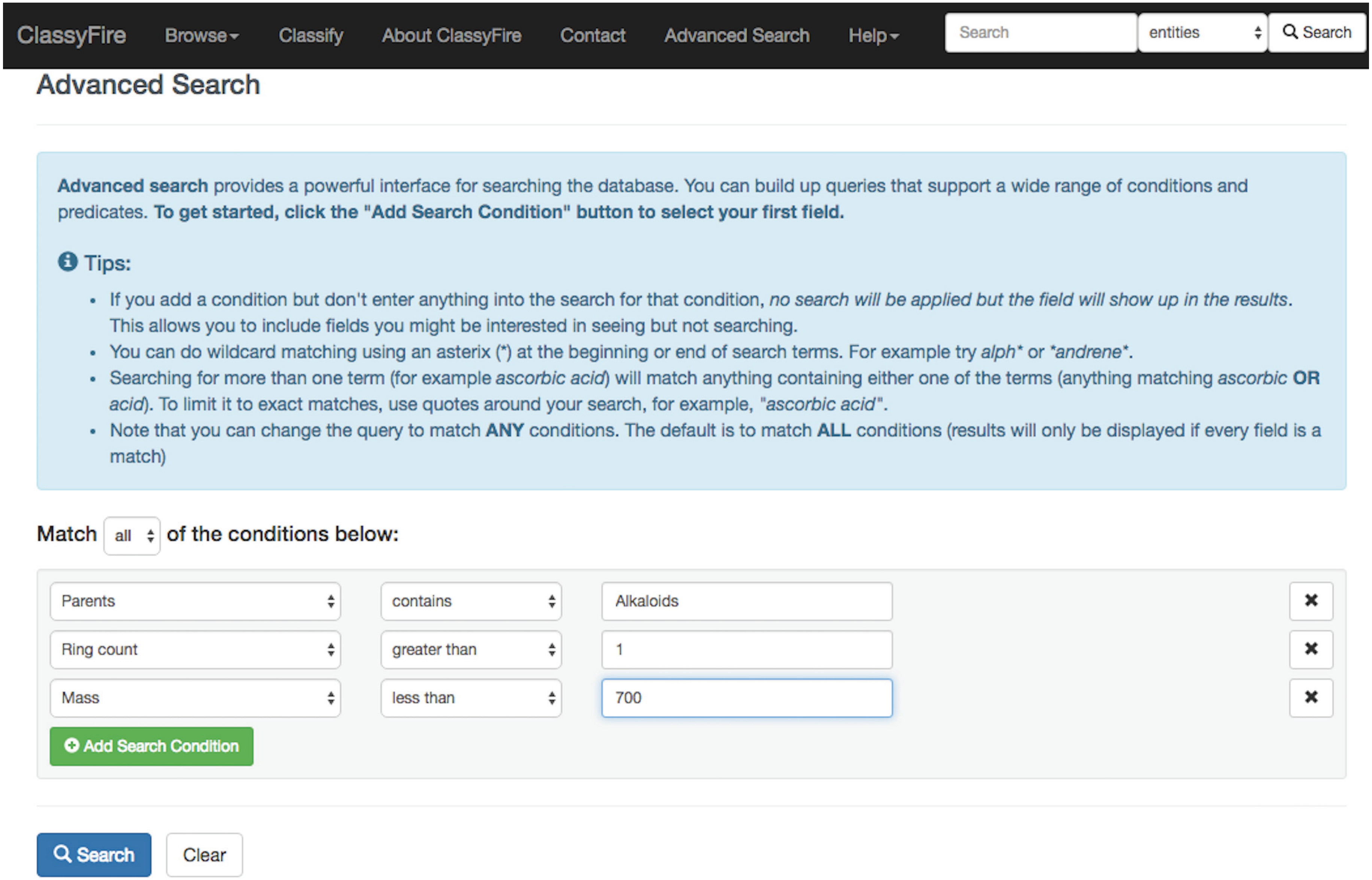
ClassyFire
Browse
Classify
About ClassyFire
Contact
Advanced Search
Help
Search
entities
Search
Advanced Search
Advanced search provides a powerful interface for searching the database. You can build up queries that support a wide range of conditions and predicates. To get started, click the "Add Search Condition" button to select your first field.
Tips:
If you add a condition but don't enter anything into the search for that condition, no search will be applied but the field will show up in the results. This allows you to include fields you might be interested in seeing but not searching.
You can do wildcard matching using an asterix (*) at the beginning or end of search terms. For example try alph* or *andrene*.
Searching for more than one term (for example ascorbic acid) will match anything containing either one of the terms (anything matching ascorbic OR acid). To limit it to exact matches, use quotes around your search, for example, "ascorbic acid".
Note that you can change the query to match ANY conditions. The default is to match ALL conditions (results will only be displayed if every field is a match)
Match all of the conditions below:
Parents
contains
Alkaloids
Ring count
greater than
1
Mass
less than
700
Add Search Condition
Search
Clear

B)

Search Results

Export

Displaying matches **1 - 30** of **85** in total

1 | 2 | 3 | Next › | Last »

SLRCCWJSBJZJBV-UHFFFAOYSA-N

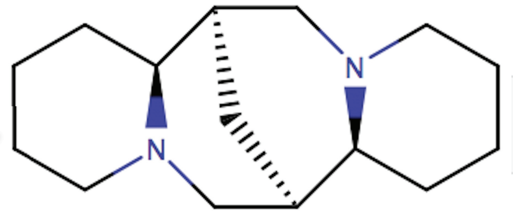

Field	Value
Parents	Quinolizidines, Chemical entities, Organic compounds, Organoheterocyclic compounds, Piperidines, Trialkylamines, Organic nitrogen compounds, Organonitrogen compounds, Amines, Tertiary amines, Azacyclic compounds, Organopnictogen compounds, Hydrocarbon derivatives, Sparteine, lupanine, and related alkaloids, Alkaloids and derivatives, Lupin alkaloids
Ring count	4
Mass	234.387
